# Bioactive Potential and Health Benefits of *Trigonella foenum‐graecum* L.: A Comprehensive Review

**DOI:** 10.1002/fsn3.70887

**Published:** 2025-09-05

**Authors:** Hassnain Akhtar, Yuosra Amer Ali, Calvin R. Wei, Reem S. Albassam, Faiyaz Ahmed, Adeela Yasmin, Musarrat Rasheed, Muhammad Sadiq Naseer, Fakhar Islam, Syeda Mahvish Zahra, Catherine Tamale Ndagire

**Affiliations:** ^1^ Department of Food Science Government College University Faisalabad Pakistan; ^2^ Department of Food Sciences, College of Agriculture and Forestry University of Mosul Mosul Iraq; ^3^ Department of Research and Development Shing Huei Group Taipei Taiwan; ^4^ Department of Community Health Sciences, College of Applied Medical Sciences King Saud University Riyadh Saudi Arabia; ^5^ Department of Basic Health Sciences, College of Applied Medical Sciences Qassim University Buraydah Saudi Arabia; ^6^ Department of Food Science and Technology Nur International University Lahore Pakistan; ^7^ Department of Clinical Nutrition Nur International University Lahore Pakistan; ^8^ Department of Food Science and Nutrition University of Sargodha Sargodha Pakistan; ^9^ Department of Nutritional Sciences Allama Iqbal Open University Islamabad Pakistan; ^10^ Department of Food Innovation and Nutrition Mountains of the Moon University Fort Portal Uganda

**Keywords:** anti‐cancer, anti inflammatory, anti‐microbial, antioxidant, diabetes, hypocholesterolemic, *Trigonella foenum‐graecum*

## Abstract

Fenugreek seeds ( *Trigonella foenum‐graecum* L.) are known for their impressive range of health benefits, thanks to their diverse array of phytochemicals. These include steroidal sapogenins like diosgenin, alkaloids such as trigonelline, as well as flavonoids, saponins, galactomannans, and polyphenols. *Trigonella foenum‐graecum* seeds contain mucilaginous fiber, which will tie bile acids and lower fat absorption and cholesterol. These bioactive constituents contribute to fenugreek's antioxidant, anti‐inflammatory, antinociceptive, antidiabetic, hypocholesterolemic, gastroprotective, antirheumatic, and antimicrobial activities. Alkaloids, 4‐Hydroxyisoleucine, and steroidal saponins have been shown to improve glucose metabolism and reduce cholesterol absorption. Additionally, specific chemical elements might excite insulin release from the B‐cells directly, resulting in a drop in blood glucose levels. *Trigonella foenum‐graecum* L. has also been found to have gastroprotective properties, antibacterial activities, and anticancer properties, and has been used to cure arthritis, lose weight, increase milk supply, and manage hyperthyroidism. Overall, fenugreek seeds represent a multi‐target natural agent with significant potential for managing metabolic disorders, inflammation, and related health conditions. Therefore, this paper aims to present the existing knowledge on the nutritional composition, bioactive potential, and health benefits in detail.

## Graphical abstract

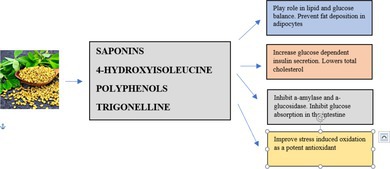

## 1 Introduction

Herbs have been used for thousands of years in many regions of the world. It is not only used as food but also as an effective medicine. They do not function in the same way as chemical medications and are not a substitute for them. Medicinal herbs are utilized by 80% of the world's population, particularly in poor nations, to treat and enhance general health (Badraoui et al. 2023). This is owing to the widespread assumption that plant‐extracted pharmaceuticals have no adverse effects and are inexpensive and easily available (Vinarova et al. 2015). *Trigonella foenum‐graecum* L. is an annual plant of the Papilionaceae‐Leguminosae family that is widely grown as a food crop in the Mediterranean regions, North Africa, India, and Yemen (Barakat et al. 2018). The spicy fragrant characteristics of fenugreek seeds are widely recognized (Niknam et al. 2021). Fenugreek has a long history in the ancient world as a cooking and medicinal plant. Its uses have been documented in Egyptian civilization, when it was used as fragrance as well as to embalm mummies (Tak et al. 2024). Many active constituents of fenugreek seed include choline, trigonelline, gentianine, and carpaine; the flavonoids apigenin, luteolin, orientin, quercetin, vitexin, and isovitexin; free amino acids such as 4‐hydroxyisoleucine; as well as arginine, histidine, and lysine, calcium and iron steroidal saponins, and so on (Jalal Aamir 2021). Various studies have been conducted to investigate the many properties of that herb and its impact on productivity. The oestrogenic characteristics of fenugreek seeds have been examined (Sreeja et al. 2010). It was studied that the blood prolactin level was increased due to its seeds (Samia et al. 2012). Fenugreek has been shown to have hypoglycemic, hypercholesterolemic, antioxidant properties, and immunomodulatory characteristics (Rebhi Hilles and Mahmood 2016). One research study described fenugreek seeds' medical usage in traditional Chinese medicine as an anti‐fungal agent, anti‐obesity, antitumor or anticancer, anti‐inflammatory, anti‐diabetic, anti‐bacterial, and hypocholesterolemic (Yao et al. 2020). Furthermore, this was found to be effective in the treatment of arthritic pain, high blood pressure, oral ulcers, diuretics, and gastrointestinal impatience (Rashid et al. 2018). Furthermore, this may be viewed as vegetarian dietary proteins that are not contained in the protein diet of animals or fish (Talib et al. 2014). Because of their haematinic value, both the leaves and seeds of fenugreek were recommended. It has been stated that fenugreek seeds consist of vitamins (A, B, and C) and a large level of iron, which boosts germination. Protein is richly found in it along with some important amino acids, like folate and ascorbate, which have nutritive and restorative characteristics, and have been shown to increase blood hemoglobin levels (Khan et al. 2018). The analgesic action of *T. foenum‐graecum* extract may be comparable to that of nonsteroidal anti‐inflammatory medications (NSAIDs). In this review, we have summarized the recent data on the pharmaceutical effects and mechanism of action of bioactive compounds present in fenugreek seeds. A detailed review on the percentage presence and specific bodily effect of each bioactive compound is presented.

## 2 Nutrients and Active Ingredients of Fenugreek

Legume plants, like fenugreek, are high‐quality foods that provide nutritional and functional benefits at a reasonable cost. Fenugreek contains a high concentration of active compounds that are beneficial to health, illness prevention, and food preservation. It is high in mucilaginous fiber and other dietary needs, and its use as a functional and nutritional food as well as a medicinal agent is possible. Its seeds are high in protein with a favorable amino acid profile, as well as lipids and biogenic components. Saponins, flavonoids, choline, carotene, essential oils containing trigonelline, and other useful components are also abundant in fenugreek seeds. The protein content of fenugreek seeds was determined to be between 235.0 (Izzo et al. 2005) to 246.0 g kg^−1^, and lipid content in the range of 40–100 g kg^−1^. Fenugreek seed contains carbohydrates ranging from 45% to 60%, the majority of which is mucilaginous fiber. There is around 20%–30% protein present, which is abundant in tryptophan and lysine. About 5%–10% fixed oils (fatty acids); alkaloids such as pyridine‐type, primarily trigonelline (0.2%–0.3%), choline (0.5%), gentianine, and carpaine; the orientin, flavonoids such as quercetin, apigenin, luteolin, isovitexin, and vitexin; as well as free amino acids such as lysine, arginine, histidine, and 4‐hydroxyisoleucine about (0.09%); Fenugreek seeds contain considerable amounts of Ca, P, Fe, Zn, and Mn, and they have significant amounts of vitamins A, B1, C, and nicotinic acid, saponins about (0.6%–1.7%); glycosides resilient steroidal sapogenins by hydrolysis (diosgenin, vamogenin, neotigogenin, tigogenin); cholesterol and sitosterol; and about 0.0015% explosive oils (n‐alkanes and sesquiterpenes), that is thought to be the cause of many of its claimed medical properties.

Both the leaves and the seeds are used as a cure for a variety of medical illnesses because of their special anti‐diabetic, blood glucose‐lowering, cholesterol‐lowering, anticarcinogenic, and antibacterial characteristics. While young seedlings are eaten as vegetables, both seeds and leaves are also used in food preparation, such as in stews in Iran, cheese flavoring in Switzerland, syrup and bitter rum in Germany, mixed seed powder for baking flat bread in Egypt, curries, dyes, and roasted seeds as a coffee substitute in Africa. An eco‐friendly plant that fixes atmospheric nitrogen is fenugreek Table 1.

**TABLE 1:** Nutritional composition of fenugreek.

Protein	246 (g kg‐1)
EAA total	304.80 (g kg‐1)
Non EAA total	576.00 (g kg‐1)
Lipid content	93.20 (g kg‐1)
Total SFAs	16.14%
Total MUFAs	15.98%
Total PUFAs	67.95%
Potassium	10 (g kg‐1)
Sodium	0.29 (g kg‐1)
Phosphors	2 (g kg‐1)
Magnesium	0.78 (g kg‐1)
Calcium	2.26 (g kg‐1)

*Source:* Ali et al. (2019); Srinivasan (2006).

## 3 Pharmacological Effects and Mechanisms of Action

**FIGURE 1:**
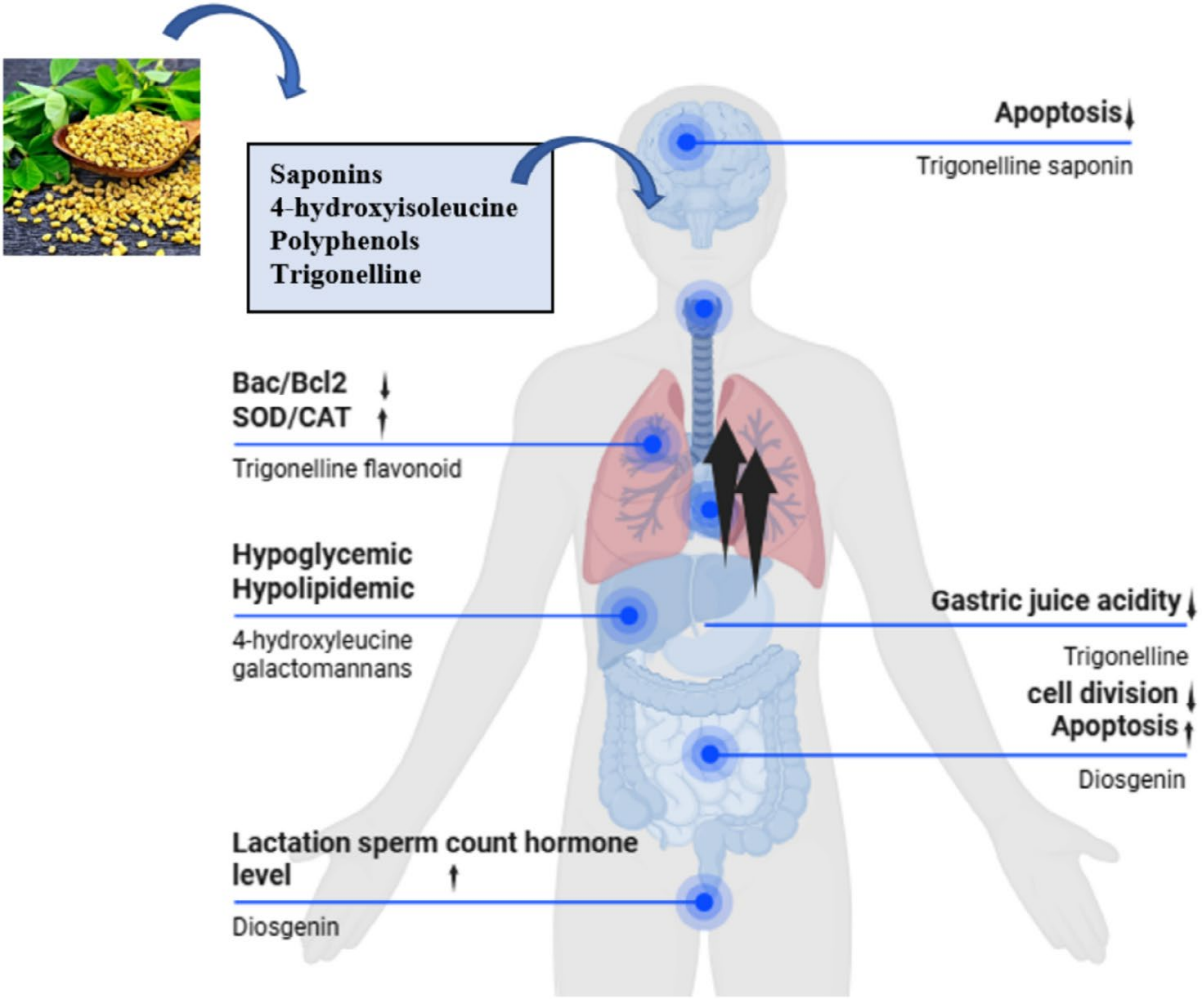
Pharmacological effect of different bioactive compounds of fenugreek.

Fenugreek has a number of pharmacological activities, including hypoglycemia, antilipidemic, and hypocholesterolemia properties. Therefore, the precise mechanism of action remains unknown (Tables 2 and 3). Fenugreek's anti‐diabetic action was assumed to be related to the creation of the mucilaginous fiber of the seeds soaked, forming a colloidal solution in the stomach and intestines, altering gastrointestinal transit and lowering glucose absorption (Tak et al. 2024). The anti‐lipidemic actions of fenugreek were considered to be related to saponin‐cholesterol formation of complexes, enhanced bile loss by fecal excretion due to saponin‐bile complexes, and therefore enhancing the liver's conversion of cholesterol to bile, and the effects of fenugreek's amino acid composition on blood cholesterol (Natural Medicines Comprehensive Database) (Kandekar, Pujari, and Thakurdesai 2022). Furthermore, this plant contains antioxidant, gastroprotective, and antirheumatic properties, as well as appetite‐stimulating properties. Histopathological investigation of the brain and liver demonstrated that the fenugreek seed aqueous extract provides considerable protection against ethanol toxicity (Belguith‐Hadriche et al. 2010; Thirunavukkarasu et al. 2003) (Figure 1).

**TABLE 2:** Potential applications of *T. foenum‐graecum* .

Category	Component/use	Description	References
Nutrients	Protein	Rich in protein, fenugreek seeds contain a protein amount that is around 23%–26%. Due to this, they serve as an excellent source of protein made from plants for those who are vegetarian or vegan in especially	Dolganyuk et al. (2023)
	Dietary fiber	Fenugreek, a plant with a high dietary fiber content, supports digestive wellness and glucose homeostasis. The insoluble as well as soluble fibers in the seeds promote the functioning of the digestive system	Awulachew (2022)
	Carbohydrates	Approximately 58% of the carbohydrate in fenugreek come in the form of in dietary fiber as well as a little proportion of starches. Carbohydrates are necessary for the production of energy	Kandekar, Pujari, and Thakurdesai (2022)
	Fats	Fenugreek seeds possess a low fat content (around 6%), but are nevertheless rich of beneficial fatty acids including oleic and linoleic acids vital to maintaining cell membrane stability	Munshi et al. (2020)
	Vitamins	Vitamins such as vitamin B6, riboflavin, and niacin, that help energy metabolism and the well‐being of the neurological in nature framework, and vitamin C, which is essential to the functioning of the immune system, all can be discovered in fenugreek	Sharma et al. (2024)
	Minerals	Iron‐rich fenugreek stimulates the production of blood cells that are red and protects from anemia. Furthermore, it contains phosphorus, magnesium, and calcium, each of which are essential for strong bones and metabolic processes	Vishwakarma et al. (2022)
Active ingredients	Saponins	Fenugreek's saponins, that additionally possess cholesterol‐lowering characteristics constitute one of the plant's numerous health benefits, that additionally include immune‐boosting and anti‐inflammatory properties	Agrawal et al. (2023)
	Alkaloids (Trigonelline)	Fenugreek includes an alkaloid termed tripeonelline, which has been established to have insulating effects and play a role control blood sugar levels, in particular for people with diabetes	Kumar and Zandi (2014)
	Flavonoids	Flavonoids, among the strong antioxidants that are found in fenugreek, shield tissues against damage from oxidative stress and reduced the risk of long‐term conditions	Singh et al. (2021)
	Diosgenin	Diosgenin is a steroidal saponin which could possess anti‐inflammatory in nature and anti‐carcinogenic properties. It helps with the production of certain hormones in addition	Tak et al. (2024)
	Mucilage	By establishing an obstacle over the intestines lining, the mucilage found in fenugreek seeds assists relieve digestive issues	Kandekar, Ramdasi, and Thakurdesai (2022)
Food uses	Spice/seasoning	A common spice in many cuisines, particularly Indian, Middle Eastern, or North African meals, is fenugreek seeds and stems	Narayana et al. (2022)
	Herbal tea	Herbal tea, which is drunk for potential health benefits, including improved digestion and control of blood sugar, is frequently produced via fenugreek seeds	Rahman and Husen (2023)
	Condiments	Curry powders, sauces, and pickle are some of the condiments that utilize ground seeds known as fenugreek. These food products have a distinctive taste owing to the seeds	Aggarwal and Bains (2023)
	Sprouted seeds	Fenugreek seeds who have sprouted are employed in sandwiches and salad. Nutrients are more readily absorbed by sprouting, thereby improving both bioavailability and their nutritional value	Ebert (2022)
	Baked goods	At times, particularly in traditional recipes, fenugreek is incorporated into bread and other baked products to enhance their flavor and nutritional value	Godebo et al. (2019)
	Health supplements	Fenugreek has been used for many different reasons, such controlling diabetes, increasing formula feeding in nursing mothers, and boosting digestion. It's available as remedies (capsules, powders, and extracts)	Awulachew (2022)
	Vegetable	Food via fresh fenugreek leaves, additionally referred to as methi, is prevalent, especially in Indian food. They are mixed with dough to make flatbreads like methi paratha, sautéed, and added to stews	Godbole (2023)

**TABLE 3:** Neuroprotective effect of fenugreek components.

Bioactive component	Main physiological effects	Mechanism of action	References
Diosgenin (steroidal saponin)	Antidiabetic (improves insulin sensitivity, lowers glucose)—hypolipidemic (lowers blood lipids)—antioxidant—anti‐inflammatory—anticancer (induces apoptosis in cancer cells)—cardioprotective	Restores pancreatic β‐cell function—downregulates hepatic gluconeogenesis—upregulates hepatic glucokinase—increases antioxidant enzyme activity—modulates adipocyte differentiation—inhibits inflammatory mediators (e.g., TNF‐α, MCP‐1, NO)—promotes apoptosis via modulation of bcl‐2 and caspase‐3	Tak et al. (2024), İşleroğlu and Türker (2023)
4‐Hydroxyisoleucine (unusual amino acid)	Antidiabetic (insulinotropic effect)—improves insulin sensitivity—regulates plasma triglycerides and cholesterol	Increases glucose‐dependent insulin secretion—stimulates Akt phosphorylation (promotes insulin signaling)—reduces MAPK and NF‐κB pathway activation (modulates inflammation)—improves lipid and glucose metabolism	Avalos‐Soriano et al. (2016), Singh and Sashidhara (2017), Ibarra et al. (2008)
Trigonelline (alkaloid)	Hypoglycemic (lowers blood sugar)—neuroprotective—anticancer potential	Inhibits sodium glucose co‐transporter‐1 (SGLT‐1) in intestinal brush border (reduces glucose absorption)—promotes pancreatic islet formation—may modulate neural and hepatocyte function	Ibarra et al. (2008), Wani and Kumar (2018)
Galactomannan (soluble fiber)	Reduces postprandial blood glucose Lowers serum cholesterol	Slows gastric emptying Forms viscous gel in the gut to delay carbohydrate absorption—modifies glucose and lipid uptake	Wani and Kumar (2018), Tewari et al. (2024)
Flavonoids & phenolics	Antioxidant—anti‐inflammatory—hepatoprotective	Scavenge free radicals—enhance endogenous antioxidant enzyme activity (e.g., SOD, GPx, GR)—inhibit lipid peroxidation—reduce MAPK and inflammatory pathway activation	Agrawal et al. (2023), Al‐Timimi (2019)
Saponins	Hypocholesterolemic (lowers blood cholesterol)—cardioprotective	Bind bile acids in the gut—increase biliary cholesterol excretion—inhibit cholesterol absorption	Rahman and Husen (2023), Wani and Kumar (2018)

## 4 Antidiabetic Effect

**FIGURE 2:**
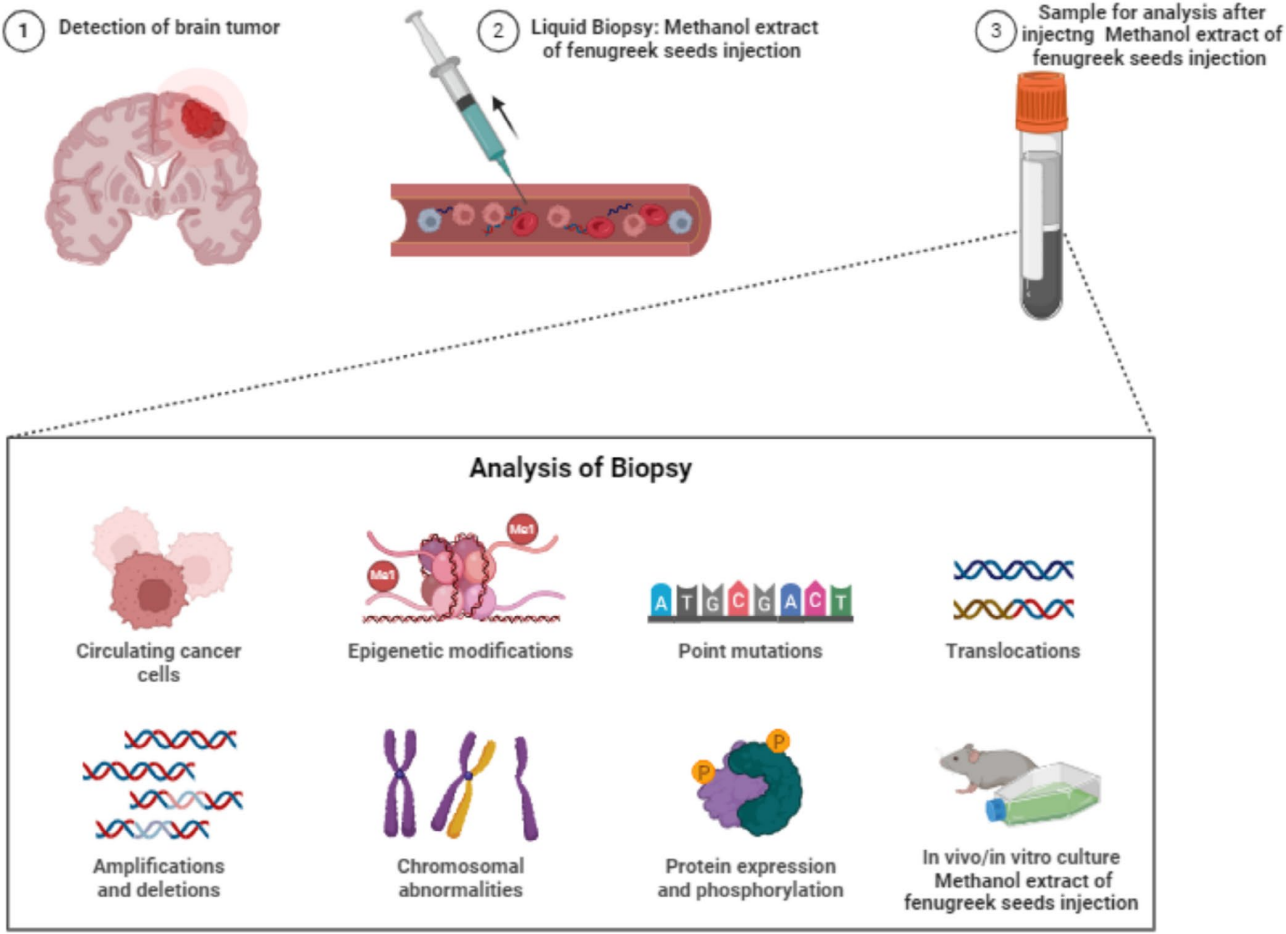
Brain cancer treatment with methanol extract of fenugreek seeds.

**FIGURE 3:**
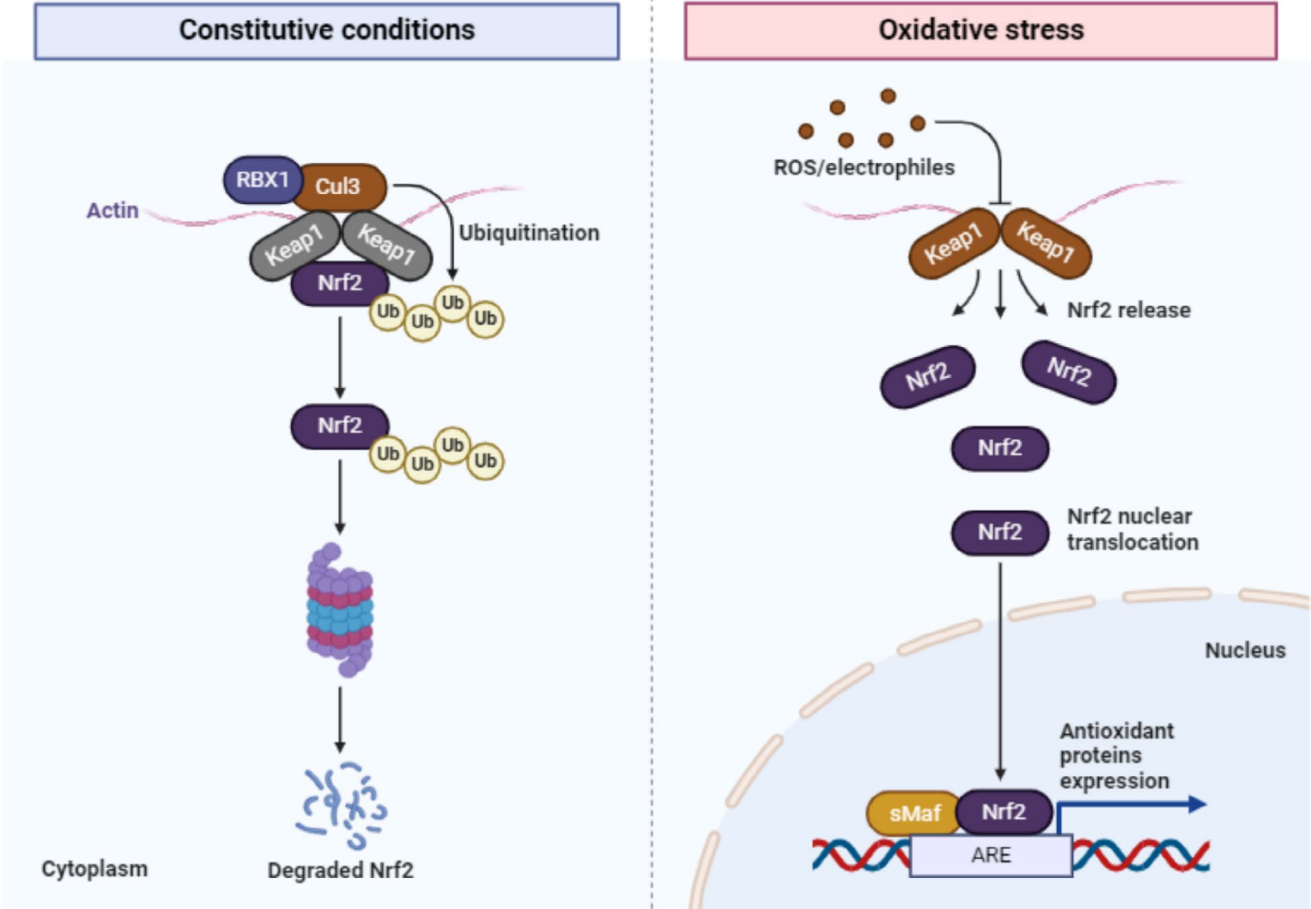
Antioxidant mechanism of fenugreek.

**FIGURE 4:**
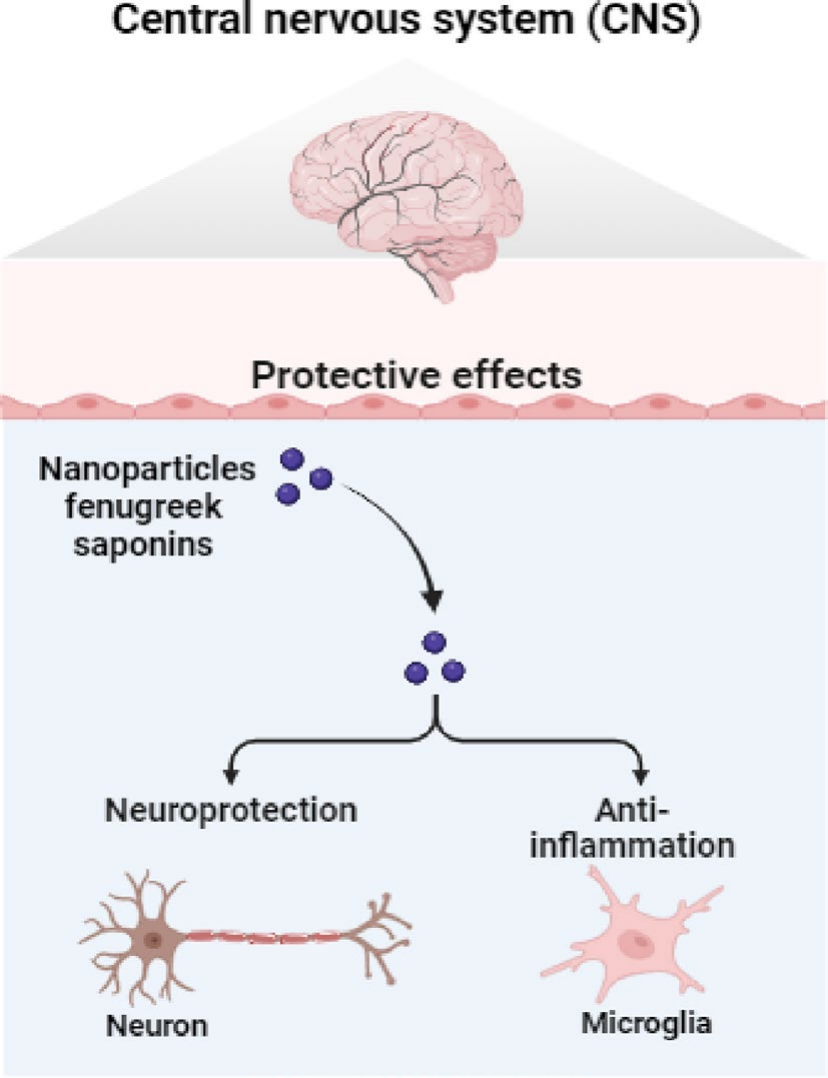
Neuroprotective properties of fenugreek component.

Unbalanced protein, carbohydrate, and lipid metabolism leads to diabetes mellitus, one of the chronic metabolic diseases. Even though there are several treatment options, including prescription medications and insulin injections, all of them have unfavorable side effects. Adopting dietary habits that not only provide a cost‐effective solution but are also packed with ingredients that promote blood sugar level regulation can help manage diabetes. The effects of fenugreek seed extract at three different dosage levels were investigated after rats were given streptozotocin to develop diabetes (Xue et al. 2007). Compared to the group of mice that only got streptozotocin, the mice fed with fenugreek grew in weight. Moreover, in comparison to the group that only got streptozotocin, the blood glucose levels dramatically fell. In their inquiry, another research team also reached a similar result. They discovered that fenugreek supplementation increased body weight growth in rabbits more than diabetes brought on by alloxan monohydrate. Following oral administration of the fenugreek seed, the levels of glucose in the plasma of both diabetic and non‐diabetic persons dropped (Abdelatif et al. 2012). Ramesh et al. (2010) investigated the effects of fenugreek seed on diabetic rats treated with alloxan. In the histological examination of the pancreas of placebo controls, the acini and cytosol of the islets of Langerhans were found to be normal. Alloxan‐induced hyperglycemia, however, severely harmed and decreased the size of Langerhans cells. In diabetic rats, the Langerhans cells may be repaired using fenugreek extract. It is feasible to extract an active ingredient with hypoglycemic properties from the crude extract (Figures 2, 3, 4). Moorthy et al. (2010) extracted GII from fenugreek seed aqueous extract in one such research. This isolated molecule was able to lower blood sugar levels when tested on sub‐diabetic and moderately diabetic rabbits. This isolated chemical outperformed even common tolbutamide. Traditional Egyptian medicine made extensive use of fenugreek as a hypoglycemic medication. Fenugreek extract was found to have a dose‐dependent capacity to suppress *α*‐amylase activity in an in vitro study by Gad et al. (2006). A second in vivo experiment came to the same conclusion and corroborated the in vitro inhibition by showing that rice's normal digestion and absorption were hindered. This shows that an insulin‐like activity was responsible for the plant extract's hypoglycemic impact. Sharma identified the effects on individuals with low blood glucose levels after ingesting the seeds or leaves. The gum derived from cooked or uncooked seeds exhibited the greatest amount of reduction, followed by the whole seed. The same study group found that regular fenugreek seed consumption significantly reduced blood levels of total cholesterol, LDL and VLDL cholesterol, and triglycerides, but had no impact on HDL cholesterol. Saponin, the amino acid 4‐hydroxyleucine, and a high‐galactomannan soluble fiber that helped boost insulin levels in rats are the main constituents that have been found to have anti‐diabetic properties (Goyal et al. 2016).

## 5 Hypocholesterolemic Effect

Animal studies have convincingly proved fenugreek's cholesterol‐lowering effect. In a typical trial, fenugreek seed fractions have been added to the meals of diabetic and hypercholesterolemic dogs. In both groups of dogs, the defatted fraction, which includes about 54% fiber and approximately 5% steroidal saponins, reduced plasma cholesterol, blood glucose, and plasma glucagon levels compared to pretreatment values. Rats have duplicated the hypocholesterolemic effect. Diabetic rats were given a fiber‐rich fraction of fenugreek, which condensed triglycerides, total cholesterol and low‐density lipoprotein (LDL) (Snehlata and Payal 2012). Furthermore, the current findings provide additional evidence for the effect of steroid saponins on plasma lipoproteins. The decrease in total cholesterol without a change in free cholesterol in VLDL‐LDL indicates a decrease in cholesterol esters. As a result, steroid saponins are thought to lower LDL (Srinivasan 2006).

Saponins are triterpenoid and steroidal aglycone plant glycosides. They are indeed a diverse collection of amphiphilic compounds with a wide variety of characteristics that are highly surface active. The majority of saponins are hemolytic, can bind cholesterol, and create stable foams (Belguith‐Hadriche et al. 2010). Thus far, research on saponins' impact on cholesterol homeostasis has focused on triterpenoid saponins produced from lucerne and steroidal saponins derived from soya bean, both of which lower cholesterol absorption in the gut. Digitonin, a steroidal saponin, has similarly been shown to prevent or decrease hypercholesterolemia in monkeys without changing HDL‐cholesterol levels. In contrast, there was no effect when rats and hamsters were fed a commercial saponin (Agrawal et al. 2023). The chemical structure and provenance of the saponin utilized in this investigation, however, were not revealed. Other saponins influence cholesterol biosynthesis indirectly by engaging with bile acids and increasing their fecal elimination. Saponin extracts may have a hypocholesterolemic effect because they block cholesterol absorption in the gut, resulting in an increase in cholesterol excretion in the stool. Saponins have been shown to form insoluble complexes with cholesterol and to restrict the availability of bile salts. These relationships may have an influence on micelle formation, reducing absorption of fat and fat‐soluble substances (Izzo et al. 2005).

Flavonoids, alkaloids, as well as saponins are found in fenugreek plant seed, but saponins are the greatest abundant (Jani et al. 2009; Kumar and Zandi 2014). Fenugreek seed contains 3.5% alkaloids, principally trigonelline, and saponin (4.8%) (Fæste et al. 2009; Jani et al. 2009). They function as cholagogic, anti‐lipidemic, and hypoglycemic medicines, and their use in the management of diabetes and hypercholesterolemia should be promoted since clinical data suggests encouraging effects in decreasing blood cholesterol levels (Izzo et al. 2005). In obese mice, fenugreek consumption reduced fat accumulation in the liver but had no impact on plasma insulin or glucose levels (Basch et al. 2003; Murlidhar and Goswami 2012).

The defensive effects of several saponin extracts had been determined due to obtaining levels of TC and TG close to normal in the livers of rats given these plant extracts. These findings are consistent with those of those who discovered that administering alfalfa saponin extract to hyperlipidemic rats dramatically decreased liver TC while increasing liver total bile acids (TBA). Saponins derived from fenugreek and licorice were shown to be more efficient than saponins isolated from asparagus and soapwort in decreasing triglyceride levels in the livers of rats given these plant extracts.

## 6 Antioxidant

The flavonoids and polyphenols existing in the fenugreek seeds add to a variety of pharmacological properties due to their anti‐oxidant capabilities. They protect the muscle, liver, brain, and heart from diabetes by boosting the glutathione reductase (GR), catalase, glutathione peroxidase, and antioxidant enzyme superoxide dismutase (SOD) (Baquer et al. 2011). In rats having diabetes, the fenugreek leaf extract lowers expressively the antioxidant system and lipid peroxidation (Annida et al. 2005). Anti‐oxidant enzymatic activity in diabetic kidneys was greatly increased by the polyphenol‐rich fractions and flavonoids of fenugreek liquid extract, defending them from morphological and functional damage (Xue et al. 2011).

Enzymes present in the liver, such as alanine aminotransferase, aspartate aminotransferase, lactate dehydrogenase, bilirubin, gamma glutamyl transferase, and alkaline phosphatase lower the glycogen level of the liver. Additionally, its drug improves lipid levels while lowering peroxidation levels, aldehyde and collagen (Kaviarasan et al. 2007).

In a Goat investigation of H_2_O_2_ and CCl_4_ persuaded liver damage, an ethanolic extract of Trigonella leaves was shown to have a considerable hepatoprotective effect, as evidenced by lowered levels of antioxidant enzymes, enzymatic & nonenzymatic. (Meera et al. 2009). In alloxan diabetic rats, dietary supplementation including fenugreek seed powder causes a decline in indicators of oxidative damage.

The TPC (total phenolic content) of the extract has been calculated as 15.1 mg gallic acid equivalent/g of dry powder. That isolate had a DPPH scavenging activity of 74.7%, which is comparable to 73.5 mM Trilox activity. In rats with bleomycin (BLM)‐induced lung fibrosis, the dried extract has been evaluated for antioxidant activity. The animals were fed a meal enhanced with 20% fenugreek seed powder or a 200 mg/kg extract.

Following bleomycin injection, the therapy was given from Days 3 to d18. The treated groups had reduced the ranges of malondialdehyde (MDA) and enhanced the ranges of total antioxidant status compared to the untreated groups (TAS). The extract and powder had only a minor effect on the bronchiolar and peribronchiolar inflammatory infiltrates, according to immunohistochemistry. Polyphenols have an anti‐inflammatory effect but have no effect on BLM‐induced structural disorganization. Due to their ability to alter the action of various enzymes, plus those involved in lipid and glucose metabolism, polyphenols have the potential to reduce liver lipids in the person. The hexane and liquid extracts of the fenugreek seeds significantly reduced COX‐2 impact, and the ethyl‐acetate extract significantly reduced COX‐1 action. The flavone 8 C glycoside extracted by water extract effectively inhibits the COX‐2 enzyme discovered by Liu et al. (2012). The urinary bladders of mice were sheltered by aqueous fenugreek extract from cyclophosphamide, which induced uterotoxicity and buthionine sulfoximine. Medication raised the action of lipid peroxidase (LPO) while lowering the action of glutathione reductase (GR), glutathione peroxidase (GP), glutathione S‐transferase (GST), and catalase (CAT) (Bhatia et al. 2006). Here on the seventh germination day, sprouted fenugreek seeds had the largest level of total phenols, which correlated to the maximum free‐radical scavenging actions (Saxena et al. 2017).

For alkaloid and phenol wrest, DPPH radical scavenging actions (0.28 and 0.36 mmol Trolox equivalent/g), metal chelating actions (0.14 and 4.03 mmol EDTA equi/g), plus FRAP standards (0.43 and 1.78 mmol Fe2 + equi/g) have been dignified. Trigonelline and phenol significantly suppressed the dipeptidyl peptidase 4 enzyme, which has been linked to diabetes progression. It was discovered that the phenol extract was four times more effective than the alkaloid extract (Sreerama and Rachana 2019).

Furthermore, it was divulged that Greek hay elevated the ability to scavenge hydroxyl radicals, signifying that it possessed strong antioxidant properties (Guardiola et al. 2018). Fenugreek seed nutrition improves tolerance to hunger and hypoxia in *Oreochromis niloticus* (Basha et al. 2018). According to another study, Greek hay has a considerable rise in phenolic antioxidants (Randhir et al. 2004). Furthermore, the antioxidant properties of fenugreek and garlic boosted glutathione blood levels and hepatic, as well as vitamin C levels in the liver and heart.

## 7 Anti‐Cancer

In vitro, fenugreek seeds (crude extracts) combined with diosgenin demonstrated potent anti‐cancer activity in a variety of cancer cell lines. Its effect was most obvious in cell lines from myeloblastic leukemia, breast cancer, prostate cancer, colon carcinoma, and esophageal cancer. It shows significant therapeutic implications for esophageal cancer and breast cancer. A handful of ex vivo experiments have shown that diosgenin with fenugreek seed extracts has anti‐cancer characteristics. Alcoholic extracts of these seeds displayed in vitro cytotoxicity against several human tumor cell lines, including neuroblastoma, IMR‐32, and human colorectal adenocarcinoma cancer cell lines. T‐cell lymphoma (TCP), B‐cell lymphoma (BCL), thyroid papillary carcinoma (FRO), and breast cancer cell lines were all shown to be preferentially cytotoxic to fenugreek aqueous extracts (MCF7) (Alsemari et al. 2014).

An ethanolic extract of fat‐free fenugreek seeds prevents the development of Ehrlich ascites cancer in rats (EAC). When the extract was administered intravenously, tumor cell growth was reduced by 86% and 94%, respectively, compared to control mice for 7 days before EAC inoculation (Singh et al. 2021). When the extract was administered after the inoculation, it inhibited tumor development by 70%. In the absence of caspase 8 or 3, as well as p53 and FADD, fenugreek methanolic extracts (FME) caused apoptosis in breast cancer cells (Al‐Shatwi et al. 2013). In a time and dose‐dependent manner, a chloroform‐based extract of fenugreek seed may significantly decrease the viability of MCF‐7 breast cancer cells by triggering apoptosis related to improved articulation of Fas, Bax, FADD, Bak, and Caspase 3, 8, 9, p53 (Khoja et al. 2011). In rats with 7,12‐dimethylbenz(a)anthracene‐induced mammary hyperplasia, the isolate was also found to promote apoptosis in breast cancer cell lines (Amin et al. 2005). The survival of human breast cancer cell lines ZR‐75‐1 and T‐47D was lowered by eight times when a decoction of Greek clover seed was used. Immunohistochemistry revealed many cellular abnormalities, cell cycle issues, and similar apoptosis traits (Vígh et al. 2016).

Fenugreek seed extract reduced colon cancer LPO and incidence in mice treated with DMH. A meal comprising fenugreek seed extract reduced colon tumor LPO and incidence in mice treated with DMH while increasing SOD, GST, GPx, and catalase activities in the liver (Devasena and Menon 2007). In cultured murine melanoma B16F1 cells and THP‐1 cells, methanolic extracts of fenugreek seed inhibited the yield of phorbol‐12‐myristate‐13‐acetate‐induced inflammatory cytokines like tumor necrosis factor (TNF)‐a (Kawabata et al. 2011). In mice, a methanol extract of fenugreek seeds protected skin tumors caused by DMBA and TPA (Chatterjee et al. 2012).

Diosgenin reduces invasion and metastasis of human prostate cancer PC‐3 cells via reducing matrix metalloproteinase production, according to Chen et al. (2011). In HepG2 cells, fenugreek seed oil is known to be cytotoxic, and in hepatocellular carcinoma cells, it caused apoptosis via the mitochondrial route in a concentration‐dependent manner. The induction of apoptosis was discovered to be linked to ROS production and mitochondrial membrane potential (Al‐Oqail et al. 2015).

As a result, diosgenin and fenugreek have appeared as viable cancer treatments for breast, colon, and liver cancers. Because diosgenin is a natural substance, it appears to be much safer than curcumin and may not cause bioavailability concerns. Due to apoptosis induction and enhanced expression of pro‐apoptotic genes, fenugreek has been demonstrated to have anticancer properties against MCF‐7 human enshrined breast cells (Khoja et al. 2011). Some other studies demonstrated that extract of fenugreek improved cell viability by increasing LDH, caspase‐3, and caspase‐6 activity in Mcf7 human breast and pancreatic (AsPC‐1) cells (Abas and Naguib 2019). By causing cell death, decreasing the expression of mutant p53, and elevating p21, treatment with 10–15 g/mL fenugreek extracts for 72 h reduced the development of breast and pancreas cancer cell lines (Shabbeer et al. 2009).

## 8 Anti‐Inflammatory

Fenugreek has a long history of use in numerous nations as a traditional medicine to treat inflammation and its side effects, including Iran, southern India, and Africa. The key chemical substances causing the anti‐inflammatory action include alkaloids, saponins, and flavonoids. Sharififara et al. (2009) investigated the in vivo effects of the methanol extract using a method based on cream. Inflammation brought on by carrageenan resulted in edema in Wistar rats, and both intraperitoneal medication and topically applied cream had anti‐inflammatory effects. Kawabata et al. (2011) investigated the effects of anti‐inflammatory and antimelanogenic agents in an in vitro setting utilizing the human monoclonal cell line (THP‐1). Inflammatory cytokines, including IL‐1, IL‐6, and TNF, were generated utilizing phorbol myristate acetate. TNF‐*α* was not produced when fenugreek extract was used as a solvent system with methanol. The extract was further processed to separate the bioactive components, such as saponin, as well as two other compounds that were also shown to inhibit TNF‐*α* and other cytokines, such as IL‐1 and IL‐6. Concentration impacted the actions of inhibitors (Singh et al. 2021).

In contrast to in vitro experiments, in vivo studies revealed distinctive outcomes. When fenugreek was administered orally, it was shown that the levels of TNF‐*α* protein in the blood and liver of fat rats increased. This demonstrates how fenugreek seed inhibits the synthesis of TNF. Through a number of intricate procedures, including digestion, absorption, and metabolism, the drug ingested orally may have an impact on live biological mediators. Fenugreek's antagonistic effects on TNF‐*α* production may be explained by the variations between in vitro and in vivo systems. A part of another study conducted by Sumanth et al. (2011) examined how anti‐inflammatory medicines may contribute to the onset of lupus (Goyal et al. 2016).

According to the ulcer index, fenugreek seeds' aqueous extract has an anti‐ulcer effect. The extract's antiulcer properties might be attributed to its well‐known antioxidant activity. *T. foenum‐graecum* leaves also have anti‐inflammatory and anti‐pyretic effects in addition to semillas (Sumanth et al. 2011). Similar to Esto, Ravichandiran, and Jayakumari used aqueous extracts of fenugreek seeds and leaves to study the anti‐inflammatory efficiency of an identified bioactive component in both in vivo and in vitro conditions. It was established that the aqueous extract of fenugreek leaves and the chloroform fraction of the seed both have anti‐inflammatory activities. In a recent study, fenugreek was shown to inhibit macrophage penetration into adipose tissue. Additionally, there was a decrease in inflammatory gene mRNA expression. The antioxidant properties of fenugreek may hasten the healing of rats with posterior neck lesions (Goyal et al. 2016).

## 9 Anti‐Microbial

Antimicrobial effect of fenugreek seeds is well documented, for example, Al‐Timimi (2019) evaluated the antibacterial activity of fenugreek seed extract on six pathological bacteria strains, which were specified through conventional biochemical tests using the Vitek2 automated system and diffusion agar method. Results showed that the highest activity of the extract of the seed was found on *Staphylococcus aureus* and *Pseudomonas aeruginosa* (22 and 17 mm diameter of inhibition zones), respectively. In another study, it was observed that the ethanolic extract of fenugreek seeds was the most active against *Escherichia coli* with the highest zone of inhibition of 14 mm, followed by *Klebsiella pneumoniae* with 13 mm, while *S. aureus* and *P. aeruginosa* had 12 mm each zone of inhibition open well diffusion and paper disc method at 10, 20, 40, 50, and 100 μL concentration (Raji‐Idowu 2023).

Fenugreek seeds also contain silver nanoparticles that have antibacterial activity against both gram‐positive and gram‐negative bacteria. The ultrasound‐aided nanoparticles outperformed magnetically mixed nanoparticles in terms of resistance, antibacterial activity, and antioxidant capacity. Studies have been done on the AgNP generated *by T. foenum‐graecum* L. seed extract's antibacterial and anticancer processes. The outcomes demonstrated that the MIC of the AgNP for *Aspergillus flavus*, *Trichophyton rubrum*, and *Trichoderma viridiae* was 250 μg mL^−1^ (Deshmukh et al. 2019). Pathogenic bacteria treated with AgNP showed a more effective antibacterial mechanism. The Kirby‐Bauer technique was used to evaluate the nanoparticles used in biosynthesis for their antibacterial properties. The TF‐TiO2 nanoparticles severely inhibited the growth of all the tested microorganisms (Deshmukh et al. 2019).

Due to the occurrence of many phyto‐chemicals, fenugreek showed significant antibacterial actions counter to pathogenic bacterial strains ( *Bacillus cereus* and *Serratia marcescens* ) as well as harmful fungal strains (*T. viride*), including a wide zone of inhibition (Dharajiya et al. 2016). Fenugreek has been shown to have antibacterial characteristics in contradiction to Gram −ve *P. aeruginosa* as well as Gram +ve *S. aureus* bacteria (Al‐Timimi 2019). Alkaloids, flavonoids, tannins, saponins, steroids and terpenoids, either alone or in combination, may contribute to fenugreek's antibacterial properties (Khursheed et al. 2012).

## 10 Neuroprotective Effect

Neuropathic pain is one of the most prevalent neurological diseases, and empirical data suggests that inflammatory cytokines and microglial cells play a significant role in the genesis of the disease. Researchers have identified the potential use of medicinal plants in the treatment of neurological illnesses using animal models. In this line, fenugreek has also been researched as a possible therapeutic plant for the treatment of neurological disorders. Fenugreek extracts' bioactive ingredients have also shown promise in reducing the risk of a number of neurological diseases. Numerous studies have demonstrated the efficacy of fenugreek components in the treatment of Parkinson's disease, Alzheimer's disease, and depression. For instance, Khalil et al. (2016) found that feeding rats fenugreek saponins (0.05%–2.0%) with their meals for 35 days showed neuroprotective benefits via reducing acetylcholinesterase (AChE) activity and cell death (Khalil et al. 2016). Similarly, Bin‐Hafeez (2003) investigated the neuroprotective effects of 5% fenugreek seed powder against aluminum chloride‐induced neurotoxicity over the course of 4 weeks in a rat model. Significant neuroprotective effects were seen with seed powder of fenugreek (Bin‐Hafeez 2003). Using ethanol extract of fenugreek to stop the action of the MAO (monoamine oxidase) A and B inhibitor clorgyline, Garcia‐Miralles et al. (2016) found that the extracts were helpful at reducing depression. The neuronal transmission was enhanced as a result. The dosages of the therapies ranged from 100 to 500 mg per kilogram (Garcia‐Miralles et al. 2016). Furthermore, it has been said that Trigonella (100 mg/kg) suppresses rotatory behavior and raises neuronal MDA (malondialdehyde) and SNC (substantia nigra compact) levels, hence reducing the incidence of Parkinson's disease (Foltynie and Kahan 2013). Wang et al. (2019) looked into the processes underlying the fenugreek flavonoids' antidepressant effects using animal models. The outcomes demonstrated that by favorably modifying the pathways and expression of proteins and enzymes, fenugreek flavonoids significantly reduced the abnormal behavior. Positive effects have also been noted on the activities of other neurotransmitters, such as decreased MAO activity (Wang et al. 2019). These findings contribute to the expanding body of evidence demonstrating the potent neuroprotective properties of fenugreek components.

## 11 Limitation

Some preparation steps, like excessive grinding or exposure to air, can lead to oxidation of sensitive compounds, reducing efficacy. Additionally, some bioactives are bound in the plant matrix, requiring enzymatic or chemical treatment to be released efficiently (İşleroğlu and Türker 2023). Conventional methods often lack selectivity, co‐extracting non‐targeted substances alongside desired bioactives. This can dilute pharmacological potency and complicate purification. Emerging technologies like ultrasound‐assisted and supercritical CO_2_ extraction can speed up processing but are sometimes costly or require specialized equipment (Niknam et al. 2021).

## 12 Conclusion

*Trigonella foenum‐graecum* L. (fenugreek) is of great multipurpose use in both nutritional makeup and bioactivity. Rich in dietary fiber, proteins, vitamins, and minerals, it harbors numerous bioactive compounds: saponins, flavonoids, and alkaloids. Fenugreek has been put to extensive examination due to its properties in lowering levels of blood glucose and cholesterol, besides antioxidant, anti‐inflammatory, and antimicrobial properties, etc. With regard to metabolism improvement and improved glucose and lipid profile, fenugreek holds very good natural repute as management in diabetic mellitus and cardiovascular diseases. Its antioxidant property is also associated with a state of reduction during oxidative stress and aging related to chronic disease. The bioavailable bioactive constituents, diosgenin and trigonelline, can exert synergism, enhancing their efficacy, therapeutically speaking, acting mostly on their management, regulation of hormonal management, and carcinoma control. Overall, fenugreek is a promising functional food for applications in enhancing human health and well‐being.

## 13 Future Recommendations

Standardization of the methods of extraction to obtain maximum yield and stability of the bioactive components in fenugreek is an area in which future research should be directed. This will need clinical trials in order to set effective doses that are safe for long‐term consumption by humans. Another promising avenue of investigation is in personalized nutrition and nutraceutical formulations containing fenugreek. Studies aimed at investigating incorporation into fortified foods, beverages, and pharmaceutical preparations will improve acceptability and accessibility. Advanced studies on the synergistic effects of fenugreek bioactives with other functional ingredients might bring innovative solutions to chronic diseases. Second, optimization of agronomic practices for increasing the yield and bioactive content of fenugreek under various environmental conditions is required. Lastly, attention to sustainable cultivation and processing methods ensures that fenugreek is going to remain a valuable resource for health around the globe.

## Author Contributions

**Hassnain Akhtar:** resources (equal), writing – original draft (equal). **Reem S. Albassam:** methodology (equal), writing – review and editing (equal). **Musarrat Rasheed:** software (equal). **Muhammad Sadiq Naseer:** validation (equal). **Yuosra Amer Ali:** methodology. **Calvin R. Wei:** validation. **Faiyaz Ahmed:** visualization and methodology. **Fakhar Islam:** data curation. **Syeda Mahvish Zahra:** software. **Catherine Tamale Ndagire:** formal analysis.

## Ethics Statement

The authors have nothing to report.

## Consent

All authors are willing for publication of this manuscript.

## Conflicts of Interest

The authors declare no conflicts of interest.

## Data Availability

The datasets generated, used, and/or analyzed during the current study are available from the corresponding author on reasonable request.
